# External fixation versus reverse shoulder arthroplasty for proximal humerus fractures in the elderly: a retrospective comparative study

**DOI:** 10.1007/s00402-026-06286-4

**Published:** 2026-04-06

**Authors:** Antonio Vadalà, Cristiano Benelli, Francesco Suraci, Benedetto Carta, Giorgio Baldassari, Nicola Maffulli

**Affiliations:** 1 Orthopaedic Unit, S. Giovanni Evangelista Hospital, Via Antonio Parrozzani, 3, RM 00019 Tivoli, Italy; 2 Orthopaedic Unit, S. Andrea Hospital, University of Rome Sapienza, Via di Grottarossa 1035-1039, 00189 Rome, Italy

**Keywords:** Proximal humerus fractures, External fixation, Reverse shoulder arthroplasty, Comparative study, Geriatric trauma, Range of motion

## Abstract

**Background:**

The optimal management of displaced proximal humerus fractures (PHFs) in the elderly remains a subject of ongoing debate. This study aims to compare the clinical and functional outcomes of biologically driven external fixation (EF) versus functional joint replacement via reverse shoulder arthroplasty (RSA) in patients aged 65-80 years.

**Methods:**

A retrospective comparative study was conducted on patients with displaced Neer two- or three-part PHFs treated between 2015 and 2022. The final analysis included 67 patients: Group A (EF; n=34) and Group B (RSA; n=33). Clinical and functional outcomes were quantified using the Constant-Murley Score (CMS), Simple Shoulder Test (SST), American Shoulder and Elbow Surgeons (ASES) score, Disabilities of the Arm, Shoulder and Hand (Quick-DASH) score, and Range of Motion (ROM) assessment. Patient satisfaction was evaluated via a 5-point Likert scale.

**Results:**

Both cohorts were homogeneous regarding mean age (72.2 ± 3.5 vs. 73.4 ± 3.8 years). Group A demonstrated significantly shorter mean operative times (48.2 ± 10.5 vs. 92.4 ± 15.2 min; p < 0.001) and a lower requirement for blood transfusions (0% vs. 12.1%; p < 0.05). At the final follow-up, both cohorts achieved comparable CMS (58.4 ± 8.2 vs. 55.2 ± 10.1; p = 0.42), SST (7.6 ± 1.5 vs. 7.0 ± 1.4; p = 0.38), and ASES scores (69 ± 7.8 vs. 65 ± 9.4; p = 0.51). Group A exhibited significantly superior external rotation (60.9° ± 9.2° vs. 46.5° ± 10.1°; p < 0.01) and a trend toward better internal rotation (49° ± 16.2° vs. 42° ± 17.1°; p = 0.09). Subjective satisfaction was comparable (Likert score: 3.2 ± 0.8 vs. 2.9 ± 0.9; p = 0.16). The overall complication rate was 20.6% in Group A and 12.1% in Group B (p = 0.51).

**Conclusions:**

Both EF and RSA are effective for managing PHFs in the elderly, yielding similar functional outcomes. However, EF represents a significantly less invasive, "bio-friendly" alternative, offering shorter surgical duration, no transfusion risk, and superior restoration of physiological rotations. The preservation of native anatomy and high patient satisfaction support EF as a viable treatment option in the geriatric population**.**

**Level of Evidence:**

Level III, Retrospective Comparative Study.

## Introduction

Proximal humerus fractures (PHFs) represent a significant clinical challenge as prevalent low-energy injuries in the geriatric population, particularly among osteoporotic women over 60 years [1–4]. Despite their frequency, the optimal management of these fractures remains a subject of ongoing debate [1–4]. Therapeutic strategies range from conservative protocols to complex surgical reconstructions, guided by both clinical presentation and radiographic parameters. While non-operative management is generally preferred when an intact “medial hinge” provides sufficient intrinsic stability [5–7], surgical decision-making for patients aged 65–80 years often remains subjective. The choice of intervention is predominantly dictated by the surgeon’s expertise, the patient’s functional requirements, and their specific comorbidity profile [4]. External fixation (EF) has emerged as a compelling minimally invasive alternative, offering the advantages of reduced operative duration, preservation of humeral head vascularity, and low incidences of infection or non-union [8–14]. Reverse shoulder arthroplasty (RSA) has gained substantial clinical traction, supported by modern implant designs and satisfactory long-term outcomes documented for up to 20 years [15]. This paradigm shift is further evidenced by the declining utilization of open reduction and internal fixation (ORIF) with plate-and-screw constructs in the elderly [16–18]. Given these evolving trends in shoulder traumatology, a direct comparison between biologically-driven fixation and functional joint replacement is imperative. The present study aims to compare the clinical and functional outcomes of EF and RSA in patients aged 65–80 years to elucidate the specific advantages and limitations inherent to each surgical philosophy.

## Materials and methods

### Study design and patient selection

This retrospective comparative study was conducted at Sant ‘Andrea Hospital (Rome, Italy) on patients treated for displaced PHFs between January 2015 and April 2022. This study was performed in accordance with the 1964 declaration of Helsinki and its later amendments or comparable ethical standards. All patients signed written informed consent forms. Inclusion criteria were as follows: age between 65 and 80 years; displaced Neer two- or three-part fractures; eligibility for surgical intervention via either EF or RSA; and a minimum clinical and radiographic follow-up of 12 months. Exclusion criteria were as follows: prior surgical interventions on the affected shoulder; acute vascular or nerve deficits; pre-existing significant shoulder dysfunction; four-part fractures or fracture-dislocations; pathological fractures; and inability to adhere to the prescribed postoperative rehabilitation protocol. The final cohort was divided into two groups based on the surgical procedure: Group A, treated with EF, and Group B, treated with RSA. The treatment allocation was not randomized but was determined by the senior surgeons’ preferred therapeutic philosophy during the study period. While EF was primarily utilized by surgeons favoring biological preservation and minimally invasive approaches, RSA was the preferred choice for the surgeons prioritizing immediate functional restoration. To ensure comparability, both groups were matched for demographic characteristics, preoperative systemic status evaluated by the Charlson Comorbidity Index (CCI) [19], and fracture classification.

## Surgical techniques: external fixation (group A)

**Fig. 1:**
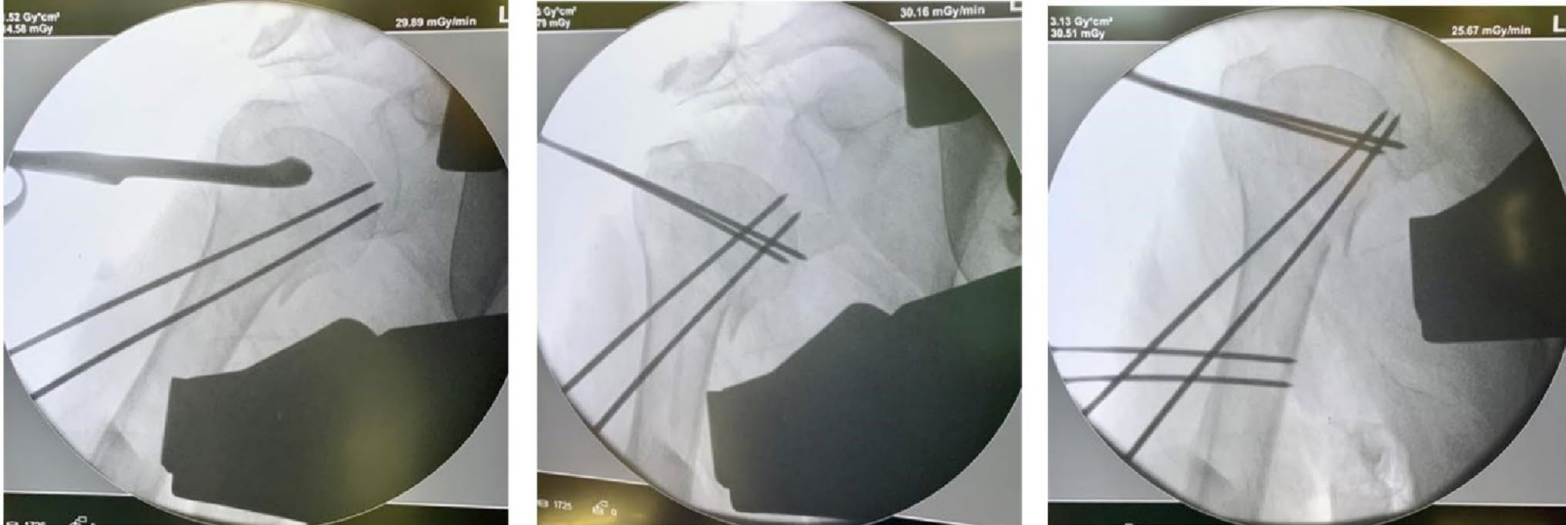
Intraoperative fluoroscopic view of external fixation (EF). Percutaneous reduction of a displaced proximal humerus fracture. Note the use of Kirschner wires (K-wires) acting as “joysticks” to achieve proper alignment before the insertion of the definitive threaded pins into the humeral head and shaft

**Fig. 2:**
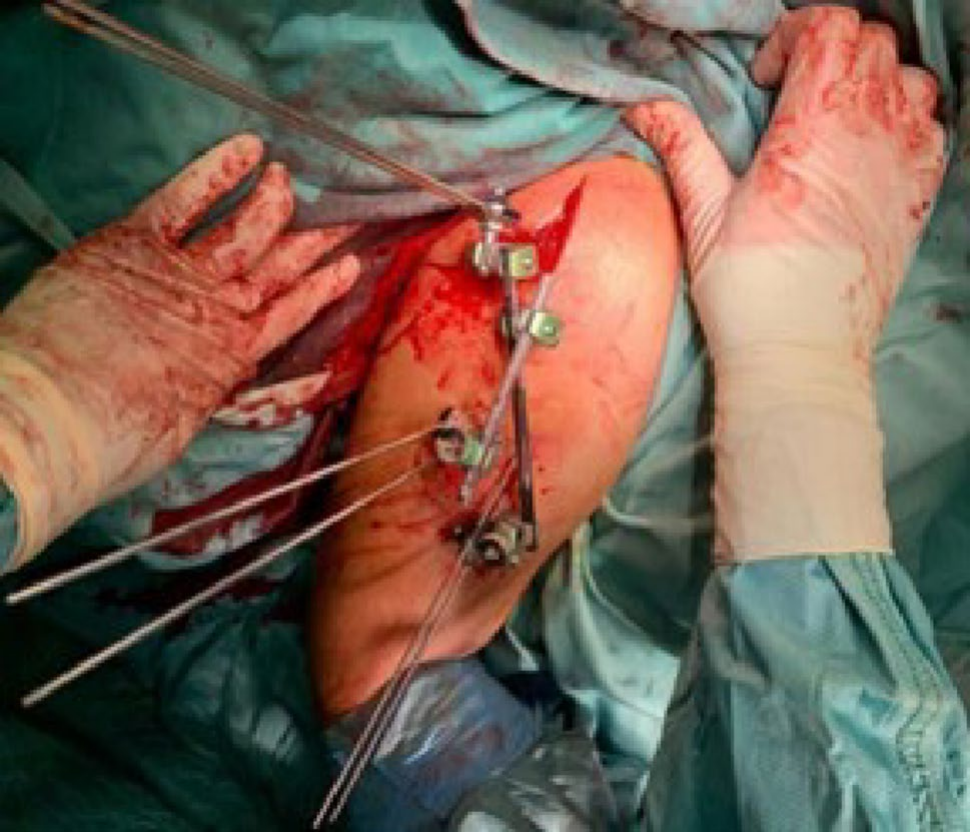
Final construct of the dedicated external fixator. Clinical photograph showing the radiolucent frame positioned according to the “safety corridor” (5 cm distal to the acromion). The minimally invasive nature of the construct allows for immediate passive mobilization while preserving the soft tissue envelope and humeral head vascularity

Patients were positioned in a beach-chair configuration under fluoroscopic guidance. A “safety corridor” was established to protect the axillary nerve (5 cm distal to the acromion) and the radial nerve (10 cm distal to the acromion) [20]. Following closed or percutaneous mini-open reduction using Kirschner wires (K-wires) as joysticks when necessary (Fig. 1), six percutaneous threaded pins (2.5–3.0 mm in diameter) were inserted to achieve bicortical shaft purchase and subchondral humeral head fixation. The construct was then locked using a dedicated radiolucent external fixator frame (Fig. 2). Postoperatively, a sling was utilized for 10 days, followed by passive physiotherapy. Active mobilization commenced immediately following pin removal at six weeks.

## Surgical techniques: reverse shoulder arthroplasty (group B)

**Fig. 3:**
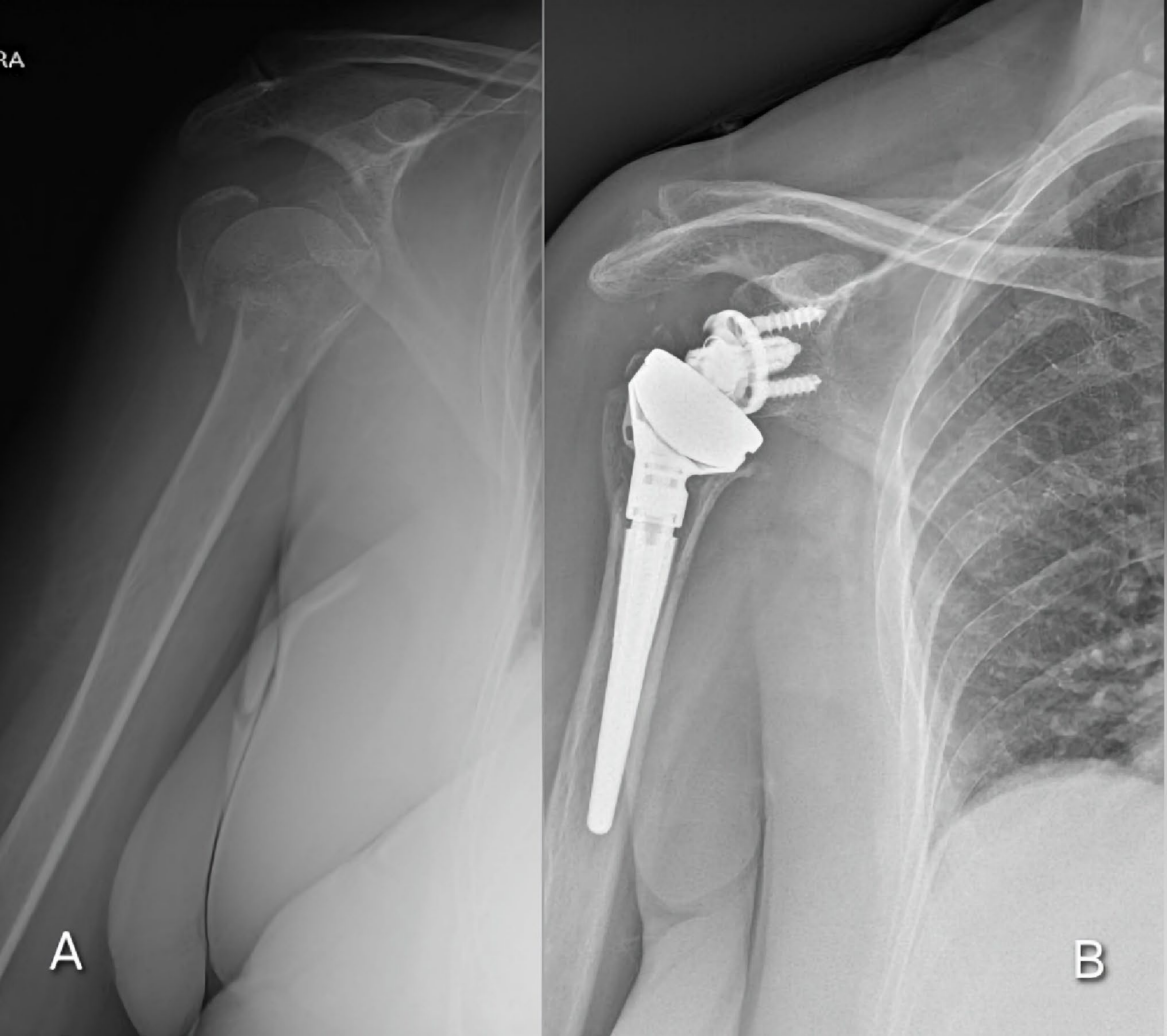
Radiographic assessment of reverse shoulder arthroplasty (RSA). **A** Preoperative anteroposterior X-ray of a complex, displaced proximal humerus fracture in a 76-year-old female patient. **B** Postoperative radiographic control showing the correct positioning of the reverse prosthesis components

A standard deltopectoral approach was employed with cephalic vein preservation. The subscapularis tendon was detached and subsequently repaired at the end of the procedure when allowed by tissue quality. Tuberosity reinsertion was performed in all cases using heavy non-absorbable sutures to optimize rotational stability. Humeral stems were either press-fitted or cemented based on intraoperative bone quality. The glenoid component consisted of a metal-back baseplate and a cobalt-chrome glenosphere with a lateralized or double-curvature design to minimize scapular notching (Fig. 3a, b). Postoperative management included a sling for three weeks, followed by progressive passive (3 weeks) and active (4 weeks) mobilization, with strengthening exercises initiated at six weeks.

## Clinical and radiographic assessment

Preoperative imaging, including standard radiographs and computed tomography (CT) scans with 3D reconstructions, was routinely performed to evaluate fracture morphology and bone quality according to the Arbeitsgemeinschaft für Osteosynthesefragen (AO) and Hertel classifications [6, 7]. Clinical follow-ups were scheduled at 1, 3, 6, and 12 months, and annually thereafter. Functional outcomes were assessed using the Constant-Murley Score (CMS) [21, 22], the Simple Shoulder Test (SST) [23], and the Disabilities of the Arm, Shoulder and Hand (Quick-DASH) score [24, 25]. Patient-reported outcome measures (PROMs) included the American Shoulder and Elbow Surgeons (ASES) score [26] and the Visual Analog Scale (VAS) for pain. Comorbidity burden was quantified via the CCI [19]. Operative times and complications were retrieved from institutional electronic health records.

### Statistical analysis

The normality of data distribution was assessed using the Shapiro-Wilk test. Quantitative continuous data (including functional scores such as CMS, ASES, and the 12-point SST) were expressed as means ± standard deviations (SD) and analyzed using the independent samples t-test for normally distributed variables or the Mann-Whitney U test for non-parametric distributions. The latter was also utilized for the analysis of ordinal data, such as the 5-point Likert scale for patient satisfaction. Qualitative categorical variables (e.g., gender, Neer classification) were compared using the Chi-square test or Fisher’s exact test, as appropriate. One-way ANOVA with Tukey’s post-hoc correction was utilized for subgroup analyses. Linear regression was performed to evaluate the correlation between clinical outcomes and CCI values. All statistical analyses were performed using IBM SPSS Statistics for Windows, version 28.0 (IBM Corp., Armonk, NY, USA). Statistical significance was defined as *p* < 0.05.

## Results

### Demographic and fracture characteristics

The final analysis comprised 67 patients: Group A (EF; *n* = 34) and Group B (RSA; *n* = 33). The two cohorts were homogeneous regarding mean age (72.2 ± 3.5 years vs. 73.4 ± 3.8 years), female gender distribution (79.4% vs. 78.8%), and traumatic mechanism. The mean follow-up was 31 ± 5.4 months for Group A (range, 12–42) and 36 ± 8.2 months for Group B (range, 12–48). Fracture distribution according to the AO classification (11 A/11B ratio) showed no significant differences between groups (27%/73% in Group A vs. 22%/78% in Group B; *p* > 0.05). Advanced CT imaging was utilized in 75% of cases to refine fracture classification (Tables 1 and 2).

**Table 1:** Patient demographics and baseline characteristics

Variable	Group A (EF) (*n* = 34)	Group B (RSA) (*n* = 33)	*P*-value
Age (years), mean (range)	72.2 ± 3.5	73.4 ± 3.8	0.18
Gender (female/male), n (%)	27 (79.4%)/7 (20.6%)	26 (78.8%)/7 (21.2%)	0.95
Side (right/left), n	17/17	20/13	0.54
Dominant limb, n (%)	22 (64.7%)	22 (66.6%)	0.93
Follow-up (months), mean (SD)	31 ± 5.4	36 ± 8.2	0.12

† : *EF*: external fixation; *RSA*: reverse shoulder arthroplasty; *SD*: standard deviation

‡ Statistical significance: * *p* < 0.05, ** *p* < 0.01, *** *p* < 0.001

§ P-values calculated via independent t-test for continuous variables and Chi-square test for categorical variables

**Table 2:** Fracture classification (Neer and AO systems)

Classification system	Group A (EF) (*n* = 34)	Group B (RSA) (*n* = 33)	*P*-value
Neer classification, n (%)			0.15
− 2-part	14 (41.2%)	8 (24.2%)	
− 3-part	20 (58.8%)	25 (75.8%)	
AO classification, n (%)			0.68
- Type 11 A (Simple)	10 (29.4%)	8 (24.2%)	
- Type 11B (Wedge)	24 (70.6%)	25 (75.8%)	

† *EF*: external fixation; *RSA*: reverse shoulder arthroplasty

‡ Statistical significance: * *p* < 0.05, ** *p* < 0.01, *** *p* < 0.001

§ P-values calculated via Chi-square test

## Perioperative data

Group A (EF) demonstrated a significantly shorter mean operative time compared to Group B (RSA) (48.2 ± 10.5 min vs. 92.4 ± 15.2 min; *p* < 0.001). Furthermore, no blood transfusions were required in the EF group, whereas four patients (12%) in the RSA group required postoperative transfusion. Baseline systemic status, quantified via the CCI [19], was comparable between cohorts (3.9 ± 1.2 vs. 4.2 ± 1.5) (Table 3).

**Table 3:** Perioperative data and complications

Variable	Group A (EF) (*n* = 34)	Group B (RSA) (*n* = 33)	*P*-value
CCI, mean (SD)	3.9 ± 1.2	4.2 ± 1.5	0.38
Operative time (min), mean ± SD	48.2 ± 10.5	92.4 ± 15.2	< 0.001***
Blood transfusion, n (%)	0 (0%)	4 (12.1%)	0.04*
Overall complications, n (%)	7 (20.6%)	4 (12.1%)	0.51
- Persistent pain, n	3	1	—
- Nerve injury, n	1†	0	—
- Infection, n	2‡	0	—
- Revision/Repositioning, n	1	0	—
- Radiographic tuberosity resorption	0	3	—

† Transient radial nerve palsy

‡ Superficial pin-site infections

§ : *CCI*: Charlson Comorbidity Index, *EF*: external fixation; *RSA*: reverse shoulder arthroplasty; *SD*: standard deviation

¶ Statistical significance: * *p* < 0.05, ** *p* < 0.01, *** *p* < 0.001. P-values calculated via independent t-test for continuous variables and Chi-square test/Fisher’s exact test for categorical variables

### Functional and clinical outcomes

At the final follow-up, both groups achieved comparable clinical and patient-reported outcomes (Table 4). No statistically significant differences were observed between Group A and Group B in mean scores for CMS [21, 22] (58.4 ± 8.2 vs. 55.2 ± 10.1; *p* = 0.42), SST [23] (7.6 ± 1.5 vs. 7.0 ± 1.4; *p* = 0.38), Quick-DASH [24, 25] (34.2 ± 9.5 vs. 38.6 ± 11.2; *p* = 0.29), ASES [26] (69 ± 7.8 vs. 65 ± 9.4; *p* = 0.51), or VAS for pain (0.59 ± 0.4 vs. 0.67 ± 0.5; *p* = 0.74). Regarding Range of Motion (ROM), anterior elevation (131.2 ± 15.4 vs. 133.5 ± 12.8; *p* = 0.81) and abduction (103.5 ± 14.2 vs. 101.4 ± 11.5; *p* = 0.77) were equivalent. However, Group A exhibited significantly superior external rotation with the arm adducted (51.4° ± 8.5 vs. 42.2° ± 7.4; *p* < 0.05) and abducted (60.9° ± 9.2 vs. 46.5° ± 10.1; *p* < 0.01). Group A also showed a trend toward superior internal rotation, which did not reach statistical significance (49° ± 16.2 vs. 42° ± 17.1; *p* = 0.09). Patient satisfaction was evaluated using a 5-point Likert scale (1: poor to 5: excellent). No statistically significant difference was found between the two groups, with a mean satisfaction score of 3.2 ± 0.8 in Group A and 2.9 ± 0.9 in Group B (*p* = 0.16).

**Table 4:** Clinical and functional outcomes at final follow-up

Outcome measure	Group A (EF) (*n* = 34)	Group B (RSA) (*n* = 33)	*P*-value
Constant-Murley Score	58.4 ± 8.2	55.2 ± 10.1	0.42
Simple Shoulder Test	7.6 ± 1.5	7.0 ± 1.4	0.38
Quick-DASH Score	34.2 ± 9.5	38.6 ± 11.2	0.29
ASES Score	69.4 ± 7.8	65.2 ± 9.4	0.51
VAS Pain (0–10)	0.59 ± 0.4	0.67 ± 0.5	0.74
Range of Motion			
Forward elevation (°)	131.2 ± 15.4	133.5 ± 12.8	0.81
Abduction (°)	103.5 ± 14.2	101.4 ± 11.5	0.77
External rotation (adducted) (°)	51.4 ± 8.5	42.2 ± 7.4	< 0.05*
External rotation (abducted) (°)	60.9 ± 9.2	46.5 ± 10.1	< 0.01**
Internal rotation	49° ± 16.2	42° ± 17.1	0.09

† : *ASES*: American Shoulder and Elbow Surgeons; *EF*: external fixation; *RSA*: reverse shoulder arthroplasty; *ROM* range of motion; *VAS*: visual analog scale

‡ Values are expressed as mean ± SD

§ Statistical significance: * *p* < 0.05, ** *p* < 0.01, *** *p* < 0.001

¶ P-values calculated via independent t-test

### Radiographic outcomes and complications

All fractures in Group A achieved primary consolidation with no instances of avascular necrosis. In Group B, no prosthetic loosening or deep infections were recorded; however, three patients (9.1%) exhibited radiographic resorption of the reinserted tuberosities. The overall complication rate was 20.6% (*n* = 7) in Group A and 12.1% (*n* = 4) in Group B (*p* = 0.51). Complications in Group A included transient radial nerve palsy (*n* = 1), superficial pin-site infections (*n* = 2), fixator mobilization requiring repositioning (*n* = 1), and persistent chronic pain (*n* = 3). Complications in Group B were limited to radiographic tuberosity resorption (*n* = 3) and persistent chronic pain (*n* = 1) (Table 3).

## Discussion

The main finding of the study is that EF provides clinical and functional outcomes comparable to RSA in patients aged 65–80, but with significantly reduced surgical invasiveness and superior restoration of external rotation. One might argue that a selection bias exists regarding bone quality and cuff integrity. However, in our series, the Hertel and AO classifications (which reflect fracture complexity) were evenly distributed between groups (p = 0.68). This suggests that the choice of treatment was not dictated by the severity of the injury, but rather by an evolving surgical paradigm that sees EF and RSA as competing, yet both viable options for the 65–80 age group. While RSA is often considered the gold standard for complex fractures, our data suggest that a biologically-driven, percutaneous approach offers a viable and ‘bio-friendly’ alternative with shorter operative times (48.2 ± 10.5 min vs. 92.4 ± 15.2 min) and a more favourable perioperative profile, characterized by the absence of blood transfusions compared to the 12.1% rate observed in the RSA group. This is consistent with the findings of Maluta et al. [14], who demonstrated that EF is a reliable option even for complex Neer 3 and 4-part fractures. The biological advantage of EF lies in the preservation of the humeral head vascularity and bone stock. By removing pins at six weeks, the native joint architecture is maintained, preserving all options for future salvage procedures if necessary [27]. No cases of avascular necrosis (AVN) were observed in our EF cohort, suggesting that the “safety corridor” and percutaneous approach effectively minimize iatrogenic vascular insult [20]. While the overall complication rate in the EF group was 20.6% (*n* = 7), the two recorded superficial pin-site infections were managed conservatively with oral antibiotics and local pin care, without requiring early fixator removal or leading to poor functional outcomes. These complications contrast favourably with the potential for deeper, more catastrophic infections associated with RSA failure, which typically necessitate more invasive revision surgeries [27]. In addition, although a learning curve for closed reduction in complex fractures exists, the procedure’s significantly shorter operative time and reduced systemic impact make it a compelling option for elderly patients with multiple comorbidities, as evidenced by comparable CCI values between our groups. A key clinical highlight is the significantly better external rotation achieved in the EF group (60.9° ± 9.2 vs. 46.5° ± 10.1). This advantage is likely due to the preservation of the rotator cuff and the 9.1% rate of tuberosity resorption observed in the RSA group, a known complication in the geriatric population characterized by osteoporotic bone [28]. Furthermore, a notable trend toward superior internal rotation was observed in the EF group (49° ± 16.2 vs. 42° ± 17.1). From a biomechanical perspective, this can be attributed to the “tissue-sparing” nature of the percutaneous approach, which avoids the tenotomy and subsequent repair of the subscapularis tendon, a mandatory step in RSA that often leads to reduced internal rotation torque [29]. Additionally, the RSA design relies on the medialization of the center of rotation, which, while beneficial for deltoid recruitment, significantly alters the tension and lever arm of the remaining rotators [30], often resulting in a less physiological range of motion compared to the native joint preservation afforded by EF [31]. Regarding patient perception, although objective scores (CMS, Quick-DASH) were equivalent, subjective patient satisfaction, assessed via a 5-point Likert scale, was slightly higher in the EF group (3.2 ± 0.8) than in the RSA group (2.9 ± 0.9; *p* = 0.16). While not statistically significant, this finding suggests that preserving the native anatomy may offer superior proprioceptive feedback and psychological comfort. The higher satisfaction in the EF group may also be linked to the less traumatic surgical experience, as evidenced by the significantly shorter operative time and lower systemic impact. However, the success of EF is heavily influenced by patient-related factors rather than fracture complexity alone. Our analysis indicates that comorbidities, summarized via the CCI, and patient adherence to rehabilitation are critical determinants of recovery. Patients with higher preoperative CCI scores tended to have poorer functional outcomes, highlighting that systemic health status contributes significantly to the overall recovery potential. Since the preoperative CCI values were comparable between the EF and RSA groups in our series, the observed functional outcomes can be more reliably attributed to the surgical approach rather than to differences in baseline systemic health. While RSA remains the indicated treatment for fracture-dislocations or head-splitting patterns, excluded from this study, our results indicate that for displaced two- and three-part fractures, the number of fragments should not be the sole factor dictating the choice of arthroplasty [32].

### Limitations

Despite its clinical relevance, this study is subject to several limitations that warrant consideration. First, the retrospective, non-randomized design inherently introduces a selection bias, as treatment allocation was dictated by clinical judgment, surgeon expertise, and individual patient profiles. Moreover, the relatively modest sample size (*n* = 67) may have limited the statistical power to detect smaller differences in clinical outcomes. Although Propensity Score Matching (PSM) was considered to further homogenize the cohorts, the relatively modest sample size precluded this statistical approach without significantly compromising the study’s power. To mitigate this, we ensured the groups were strictly homogeneous regarding key baseline variables, including age, gender, and injury mechanism, as well as systemic health status, as evidenced by the comparable CCI values. Second, the absence of a conservative management control group limits the comparison with non-operative outcomes, which have been debated in recent literature for specific fracture patterns. Furthermore, the lack of an open reduction and internal fixation (ORIF) comparison group, while reflecting the declining trend of plate-and-screw constructs in this geriatric demographic, prevents a broader analysis of all current surgical paradigms. Finally, although the ≥ 1-year follow-up provided a sufficient window to assess primary clinical stability and biological consolidation, large-scale prospective randomized controlled trials with extended follow-up are necessary to refine patient selection criteria and confirm the long-term effectiveness of the external fixation approach.

## Conclusion

The present investigation demonstrates that both EF and RSA are effective treatments for displaced proximal humerus fractures in patients aged 65–80. EF represents a significantly less invasive alternative, providing comparable functional outcomes and a highly superior restoration of external rotation (*p* < 0.01). A primary advantage of this biologically-driven approach is the preservation of the joint’s native anatomy, maintaining future surgical options if necessary. While RSA remains a reliable solution for specific indications, such as severe rotator cuff deficiency or poor bone stock, the high patient satisfaction and reduced surgical burden support the use of EF as a primary treatment option in selected elderly individuals.

## Data Availability

No datasets were generated or analysed during the current study.
